# Sphingolipids are required for exocyst polarity and exocytic secretion in *Saccharomyces cerevisiae*

**DOI:** 10.1186/s13578-020-00406-2

**Published:** 2020-03-30

**Authors:** Qingguo Guo, Tianrui Zhang, Na Meng, Yuran Duan, Yuan Meng, Dong Sun, Ying Liu, Guangzuo Luo

**Affiliations:** 1 Institute of Translational Medicine, China Medical University, Shenyang, 110122 China; 2 Department of Biochemistry and Molecular Biology, China Medical University, Shenyang, 110122 China

**Keywords:** Sphingolipid, Ceramide, Serine palmitoyl-transferase, Exocyst, Exocytosis

## Abstract

**Background:**

Exocytosis is a process by which vesicles are transported to and fused with specific areas of the plasma membrane. Although several studies have shown that sphingolipids are the main components of exocytic compartments, whether they control exocytosis process is unclear.

**Results:**

Here, we have investigated the role of sphingolipids in exocytosis by reducing the activity of the serine palmitoyl-transferase (SPT), which catalyzes the first step in sphingolipid synthesis in endoplasmic reticulum. We found that the exocyst polarity and exocytic secretion were impaired in *lcb1-100* mutant cells and in wild type cells treated with myriocin, a chemical which can specifically inhibit SPT enzyme activity, suggesting that sphingolipids controls exocytic secretion. This speculation was further confirmed by immuno-fluorescence and electron microscopy results that small secretory vesicles were accumulated in *lcb1-100* mutant cells.

**Conclusions:**

Taken together, our results suggest that sphingolipids are required for exocytosis. Mammals may use similar regulatory mechanisms because components of the exocytic secretion apparatus and signaling pathways are conserved.

## Background

Exocytosis is a cellular process of secretory vesicles transporting to specific areas of the plasma membrane [18]. Cell surface growth is ultimately performed through exocytic secretion, in which post-Golgi secretory vesicles carrying proteins and lipids are docked to and then fused with the plasma membrane [15]. The exocyst complex is composed of Sec3, Sec5, Sec6, Sec8, Sec10, Sec15, Exo70, and Exo84, which “tethers” the secretory vesicles and the plasma membrane before SNARE-mediated membrane fusion [12, 15, 18, 33, 44]. Previous studies have shown that polarity of most exocyst subunits in bud depends on exocytic secretion [1, 24, 26, 45].

Sphingolipid biosynthesis in yeast begins in the endoplasmic reticulum (ER) and is completed in the Golgi apparatus [6, 36]. Analysis of the lipid class composition indicates post-Golgi exocytic vesicles and the trans-Golgi network (TGN) contain large amount of sphingolipids, including ceramide, inositolphosphoryl ceramide (IPC), mannosyl-inositolphosphoryl ceramide (MIPC), and mannosyl-diinositolphosphoryl ceramide (M(IP)_2_C) [20, 43], suggesting sphingolipids may be involved in exocytic secretion. The mutants of several synthesis enzymes in the sphingolipids pathway have been found to impair the transport of the artificial fusion protein Fus-Mid-GFP from Golgi to plasma membrane, supporting this speculation. The elongase Elo3 which synthesizes very long chain 20–26-carbon fatty acids from C18-CoA primers, the sphinganine C4-hydroxylase Sur2 which catalyses the conversion of sphinganine to phytosphingosine, and the alkaline ceramidase Ypc1 which catalyzes both breakdown and synthesis of phytoceramide, are required for a cargo protein after synthesis at the ER throughout the secretory pathway to the cell surface [37]. Notably, it was reported that cell surface delivery of Fus-Mid-GFP is blocked in *lcb1-100* mutant, in which the activity of serine palmitoyl-transferase, an enzyme in the first step of sphingolipids synthesis, is dramatically reduced at a restrictive temperature [37]. These facts suggest that sphingolipids may be involved in exocytic secretion. However, there is no direct evidence to confirm this speculation yet.

In this study, we have investigated the role of sphingolipids in exocytosis process in the budding yeast *Saccharomyces cerevisiae*. We found that polarized localization of exocyst complex at the bud and the exocytic secretion of Bgl2 and invertase rely on the activity of serine palmitoyl transferase, the initial enzyme in sphingolipid synthesis, but not the enzymes required for ceramides. We also found that small secretory vesicles were accumulated in *lcb1-100* mutant cells, suggesting the exocytic secretion was impaired. These results suggest that sphingolipids are required for exocytic secretion.

## Results

### Activity of serine palmitoyl-transferase is required for polarized localization of exocyst

**Fig. 1:**
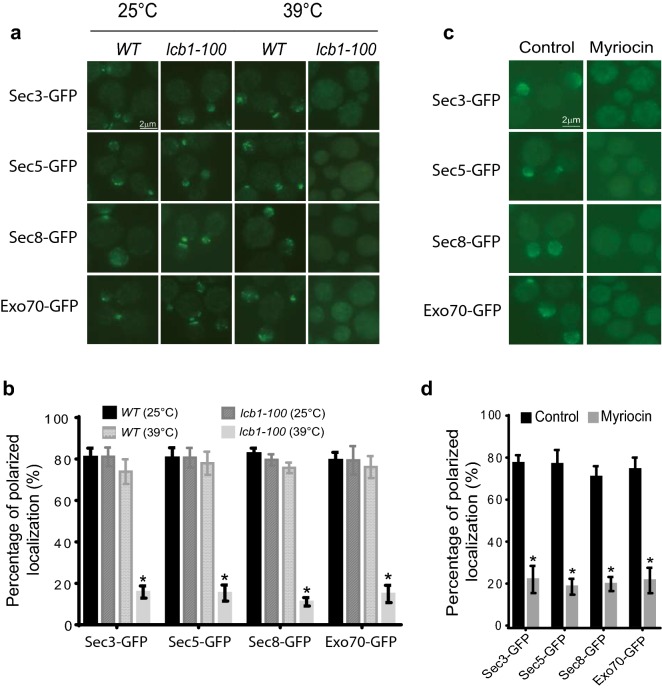
Localization of the exocyst components in *lcb1-100* mutant cells and in cells treated with myriocin. **a** The yeast strains harboring *SEC3-GFP*, *SEC5-GFP*, *SEC8-GFP* or *EXO70-GFP* on the chromosome were grown to early log phase in YPD liquid medium overnight at 25 °C. The cells were then shifted from 25 to 39 °C for 60 min before fluorescence microscopy. GFP-tagged Sec3, Sec5, Sec8 and Exo70 remained polarized in buds of wild-type cells at both 25 °C and 39 °C. The exocyst components in *lcb1-100* mutant cells were located to bud at 25 °C but dispersed in cytoplasm at 39 °C after the temperature shift. Scale bar, 2 μm. **b** Statistical analysis was performed on the percentage of polarized localization in each yeast strains at 25 °C and 39 °C in **a**. n = 3. Three independent experiments were performed. *p < 0.01. **c** Wild-type cells harboring *SEC3-GFP*, *SEC5-GFP*, *SEC8-GFP* or *EXO70-GFP* on the chromosome were grown to early log phase in YPD liquid medium overnight at 25 °C. The cells were then treated with or without 10 μg/ml myriocin for 60 min. 2 µl of the suspension was dropped onto the microslide for microscopy. Scale bar, 2 μm. **d** Statistical analysis was performed for the percentage of polarized exocyst localization in each yeast strains **a**. n = 3. Three independent experiments were performed. *p < 0.01

To explore the role of sphingolipids in exocytosis, we used the temperature sensitive mutant strain *lcb1-100.* The *LCB1* gene encodes a subunit of the serine palmitoyl-transferase, which catalyzes the first step in sphingolipid synthesis [2, 7]. *lcb1-100* mutant is therefore unable to produce sphingoid bases, ceramides, and sphingolipids at restrictive temperature [17, 27, 30, 46]. Since the delivery of exocyst subunits to polarized growth sites relies on exocytic secretion pathway, we want to know whether sphingolipids are required for polarized localization of exocyst. To test this hypothesis, the exocyst subunits, Sec3, Sec5, Sec8, and Exo70, were GFP tagged at the C-terminal of each protein on the chromosome of wild type and *lcb1-100* mutant strains, respectively. The GFP tagged exocyst subunit yeast strains were grown to early log phase at 25 °C, and then half of the cells were shift to 39 °C for 60 min. The collected cells were fixed and examined under a fluorescence microscope. As shown in Fig. 1a, b, Sec3-GFP, Sec5-GFP, Sec8-GFP, and Exo70-GFP were located in buds at both 25 °C and 39 °C in more than 80% of wild-type cells. Similarly, the GFP tagged exocyst subunits were located in buds at 25 °C in more than 70% of *lcb1-100* mutant cells. At 39 °C, however, Sec3-GFP, Sec5-GFP, Sec8-GFP, and Exo70-GFP were located in buds of less than 15% of *lcb1-100* mutant cells. The amount of the GFP tagged exocyst subunits did not differ significantly between wild type and *lcb1-100* mutant cells after shifting to 39 °C for 60 min (Additional file 1: Figure S1A). To exclude the possibility that the *lcb1-100* mutants cells are dead or dying under our experimental condition, the viability of wild type and *lcb1-100* mutants cells shifted from 25 to 39 °C for 60 min were measured. As shown in Additional file 1: figure S2, around 80% of the *lcb1-100* mutant cells were still viable after incubated at 39 °C for 60 min, suggesting that the mis-localization of exocyst components in *lcb1-100* mutant cells was not because of cell death. Consistent with previous reports, treatment at restrictive temperature for 60 min did not arrest *lcb1-100* mutant cells at early G1 phase of the cell-cycle progression [46], suggesting the polarized growth of *lcb1-100* mutant cells was not completely inhibited in such a short term. Because the bud is considered to be the growth area of budding yeast, this result indicates that sphingolipids are required for polarized localization of exocyst at the growth sites.

To confirm the role of sphingolipids in exocytosis, we inhibited the activity of SPT enzyme by treating wild type cells with myriocin to determine if exocyst is depolarized. It has been shown that myriocin specifically inhibits the activity of serine palmitoyl transferase, and has been widely used to reduce sphingolipid level in eukaryotic cells [5, 22, 27], Daquinag et al. 2012. As shown in Fig. 1c, d, myriocin did trigger a loss of exocyst in buds after cells were treated with 10 μg/ml myriocin for 1 h. The localization of Sec3-GFP, Sec5-GFP, Sec8-GFP, and Exo70-GFP was polarized in more than 72% of wild type cells without myriocin treatment. However, after treated with myriocin for 1 h, the polarized localization of these exocyst subunits was maintained only in less than 23% of the cells. The amount of the GFP tagged exocyst subunits did not differ significantly between myriocin treated and untreated cells (Additional file 1: Figure S1B). To exclude the possibility that the cells treated with myriocin are dead under our experimental condition, the viability of wild type cells treated with myriocin for 1 h were measured. As shown in Additional file 1: Figure S3, more than 90% of the cells treated with myriocin were still viable, suggesting that the mislocalization of exocyst components in myriocin treated cells was not because of cell death. It was reported that sphingolipids are required for polarized localization of actin patch [46]. To confirm the effect of myriocin on wild type cells, we measured actin organization in cells treated with 10 μg/ml myriocin for 1 h. Consistent with the previous report, the polarity of actin organization was disrupted upon myriocin treatment under our experimental condition (data not shown), suggesting the sphingolipid level was reduced in these cells. Taken together, these results confirm the critical role of sphingolipids in controlling polarized localization of exocyst complex.

### Activity of serine palmitoyl-transferase is required for exocytic secretion

**Fig. 2:**
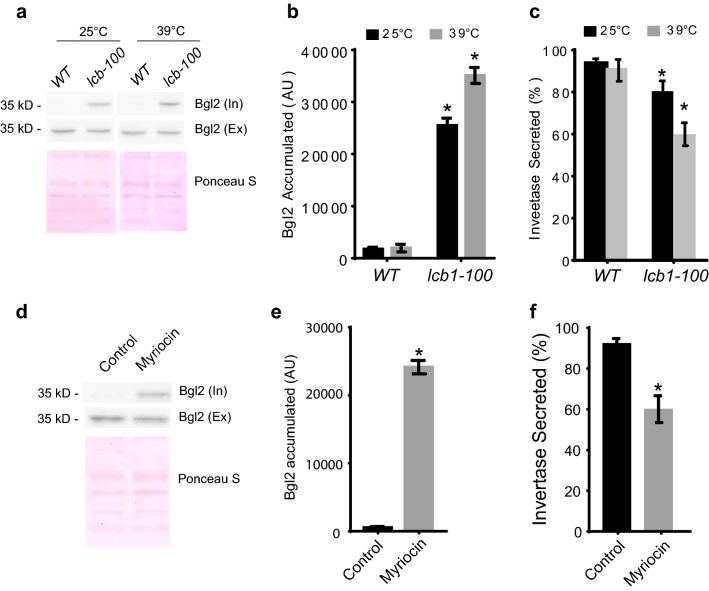
Sphingolipids are required for exocytic secretion. **a** Bgl2 secretion in the wild-type and *lcb1-100* temperature-sensitive mutant. Wild-type and *lcb1-100* mutant cells were grown to early log phase in YPD liquid medium overnight at 25 °C. The cells were then shifted to 25 °C or 39 °C for 60 min. Bgl2 secretion was analyzed by western blot using anti-Bgl2 antibody. The total protein on PVDF membranes were stained with Ponceau S as loading control. **b** Quantification of the amounts of Bgl2 accumulation in the wild-type and *lcb1-100* mutant in **a**. n = 3. *p < 0.01. **c** Invertase secretion in the wild-type and *lcb1-100* mutant cells. Wild-type and *lcb1-100* mutant were grown to early log phase in YPD liquid medium overnight at 25 °C. The cells were then shifted to 25 °C or 39 °C for 60 min. Then the invertase secretion were examined. **d** Bgl2 secretion in wild type cells treated with or without myriocin. Wild-type cells were grown to early log phase in YPD liquid medium overnight at 25 °C. Myriocin was added into the medium to a final concentration of 10 μg/ml, and the cells were incubated at 25 °C for 60 min. External and internal Bgl2 secretion was analyzed by western blot using anti-Bgl2 antibody. The total protein on PVDF membranes were stained with Ponceau S as loading control. **e** Quantification of the amounts of Bgl2 accumulation in **a**. n = 3. Three independent experiments were performed. *p < 0.01. **f** Invertase secretion in wild-type cells treated with or without myriocin. Wild-type yeast were grown to early log phase in YPD liquid medium overnight at 25 °C. Myriocin was added into the medium to a final concentration of 10 μg/ml, and the cells were incubated at 25 °C for 60 min. n = 3. Three independent experiments were performed. *p < 0.01

To determine whether sphingolipids control exocytic secretion, we have analyzed the secretion of two cargoes in wild type and *lcb1-100* mutant cells, the cell wall endo-glucanase Bgl2 and the periplasmic enzyme invertase, which are well-characterized markers for the two major classes of post-Golgi vesicles that deliver proteins to the cell surface [8, 16, 17, 23, 25, 26, 48]. As shown in Fig. 2a, b, the internal Bgl2 level in wild type cells is barely detected at both 25 °C and 39 °C. The internal Bgl2 in *lcb1-100* mutant cells at 25 °C, however, is significantly higher than that in wild type cells, and the secretion defect of Bgl2 in *lcb1-100* was exaggerated at 39 °C compared to wild type cells. Invertase represents a smaller branch of cargoes in the secretory pathway and is also used as an exocytic secretion marker as a complement to the Bgl2 secretion assay [8, 23, 26, 35, 48]. As shown in Fig. 2c, the secretion efficiency of invertase in *lcb1-100* mutant cells is significantly reduced compared to that in wild type cells at 25 °C. At 39 °C, the secretion defect of invertase in *lcb1-100* was exaggerated compared to wild type cells. The secretion defects of Bgl2 and invertase in *lcb1-100* mutant cells indicate that sphingolipids are required for exocytic secretion.

To confirm the role of sphingolipids in regulating exocytic secretion, two type of exocytic cargoes, Bgl2 and invertase, were examined in cells treated with myriocin, which can specifically inhibit activity of SPT enzyme. As shown in Fig. 2d, e, in cells without myriocin treatment, very small amount of Bgl2 was detected in cytosol. However, the amount of Bgl2 retained in the cytosol of cells treated with 10 μg/ml myriocin significantly increased compared to cells without myriocin treatment. The secretion of invertase was also measured in cells treated with myriocin. As shown in Fig. 2f, more than 90% of the invertase was secreted out of cytosol in cells without treatment. In cells treated with 10 μg/ml myriocin for 1 h, however, less than 60% of invertase was secreted out of cytosol, which is significantly reduced compared to cells without myriocin treatment. Taken together, we conclude that exocytic secretion was inhibited in cells treated with myriocin.

### Polarized localization of Sec4 was impaired in *lcb1-100* mutant cells

**Fig. 3:**
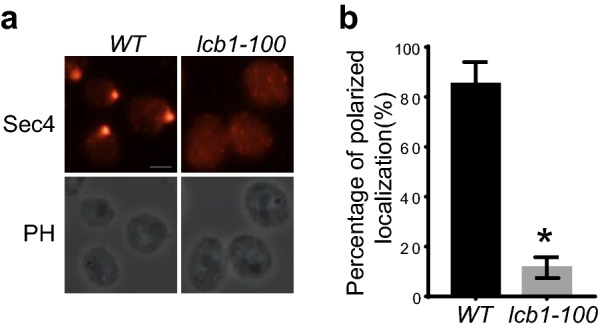
Localization of Sec4 in wild-type and *lcb1-100* mutant cells. **a** Wild type and *lcb1-100* mutant cells were grown to early log phase at 25 °C. The cells were then shifted from 25 to 39 °C for 60 min before immuno-fluorescence microscopy. Sec4 is polarized in wild type cells, while it is dispersed in *lcb1-100* mutant cells at restrictive temperature. **b** Statistical analysis was performed on the percentage of polarized localization of Sec4 in yeast strains in **a**. n = 3. Three independent experiments were performed. *p < 0.01

We have experimentally demonstrated that sphingolipids are required for polarized localization of exocyst, but its role in vesicle trafficking remains unknown. Sec4, the Rab protein residing on the post-Golgi vesicles, has been widely used as a marker by immuno-fluorescence to examine the subcellular localization of post-Golgi vesicles [9, 26, 38, 39, 41, 48]. We therefore examined the localization of Sec4 in *lcb1-100* mutant cells by immuno-fluorescence. Wild type and *lcb1-100* mutant strains were grown to early log phase at 25 °C, then shifted to 39 °C for 60 min. After antibody incubation for immuno-fluorescence, the cells were observed under the fluorescence microscope. As shown in Fig. 3, Sec4 was polarized in more than 82% of wild type cells. However, after treated at 39 °C for 60 min, the polarized localization of Sec4 was maintained only in less than 15% of the *lcb1-100* mutant cells. This result suggests that either there is no post-Golgi vesicles produced or the secretory vesicles are dispersed in *lcb1-100* mutant cells.

### Secretory vesicles were accumulated in *lcb1-100* mutant cells

**Fig. 4:**
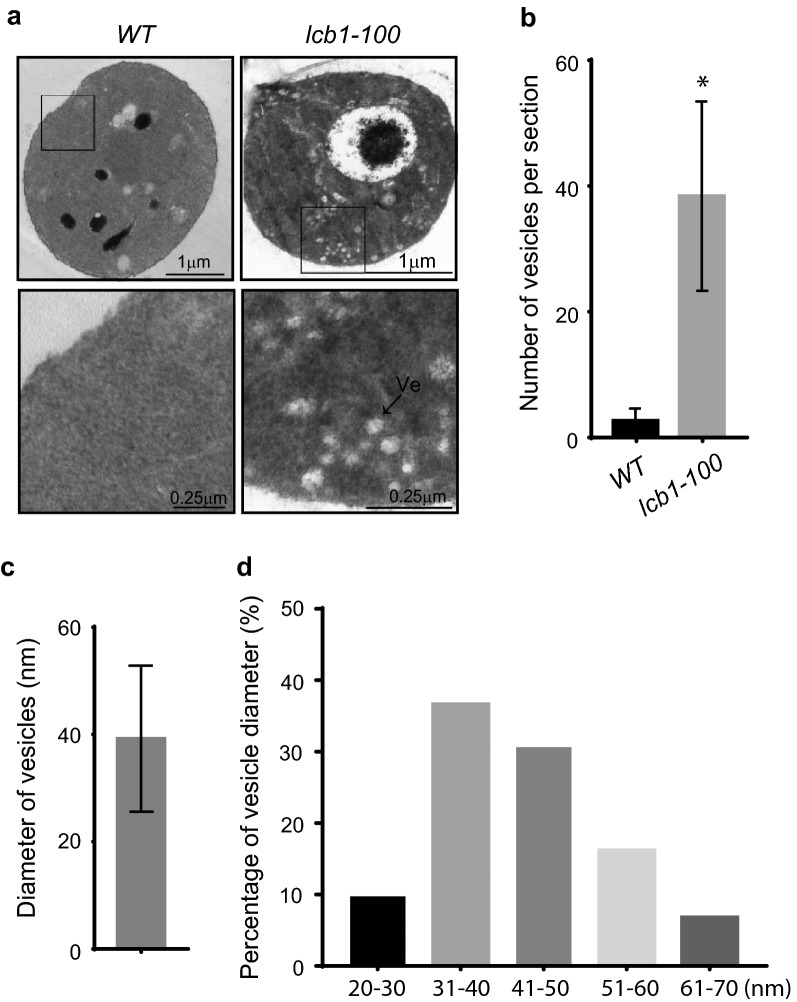
Secretory vesicles were accumulated in *lcb1-100* mutant cells at restrictive temperature. **a** Wild-type and *lcb1-100* mutant cells were grown to early log phase in YPD liquid medium overnight at 25 °C and then shift to 39 °C for 60 min. The cells were fixed, stained, and examined by thin-section electron microscopy. **b** Statistical analysis of vesicle accumulation in wild type and *lcb1-100* mutant cells in **a**. n = 15. *p < 0.01. “Ve”, vesicle. **c** Diameter measurement of vesicles accumulated in *lcb1-100*. n = 600. **d** Histogram of vesicle diameter distribution in the *lcb1-100* mutant cell shown in **c**

The events order of the exocytic secretory pathway in *Saccharomyces cerevisiae* has been determined using the temperature-sensitive secretory mutants [1, 18, 15, 34, 35, 40, 45], which confirmed that as a secretory protein matures it usually moves sequentially from the cytosol to endoplasmic reticulum (ER), Golgi, plasma membrane, and finally is exocytosed upon the fusion of post-Golgi vesicles and plasma membrane. Although we have found that sphingolipids are required for Bgl2 and invertase secretion, it is not clear how sphingolipids affect exocytic vesicle trafficking process. To answer this question, we examined the subcellular structure of wild type and *lcb1-100* mutant at restrictive temperature by electron microscopy. As shown in Fig. 4a, b, about 40 vesicles per section are accumulated in *lcb1-100* mutant, but less than 3 vesicles per section found in wild type. No elongated ER or Berkeley Body was observed in both wild type and *lcb1-100* mutant cells. We further measured the diameter of vesicles accumulated in *lcb1-100* mutant cells. As shown in Fig. 4c, d, the average diameter of vesicles in *lcb1-100* is around 40 nm and the size of most vesicles are 31–60 nm, which is very different from the size of post-Golgi secretory vesicles, 80–100 nm. It has been suggested that the small vesicles with size of 40–60 nm may originated from either ER or Golgi complex [34, 35], and may mediate transfer of material from the ER to the Golgi [35]. However, we can not conclude that sphingolipids mediate the step of ER to Golgi during exocytic secretion. Further experiments are needed to confirm this speculation.

### Ceramides and complex sphingolipids are not required for exocytosis

**Fig. 5:**
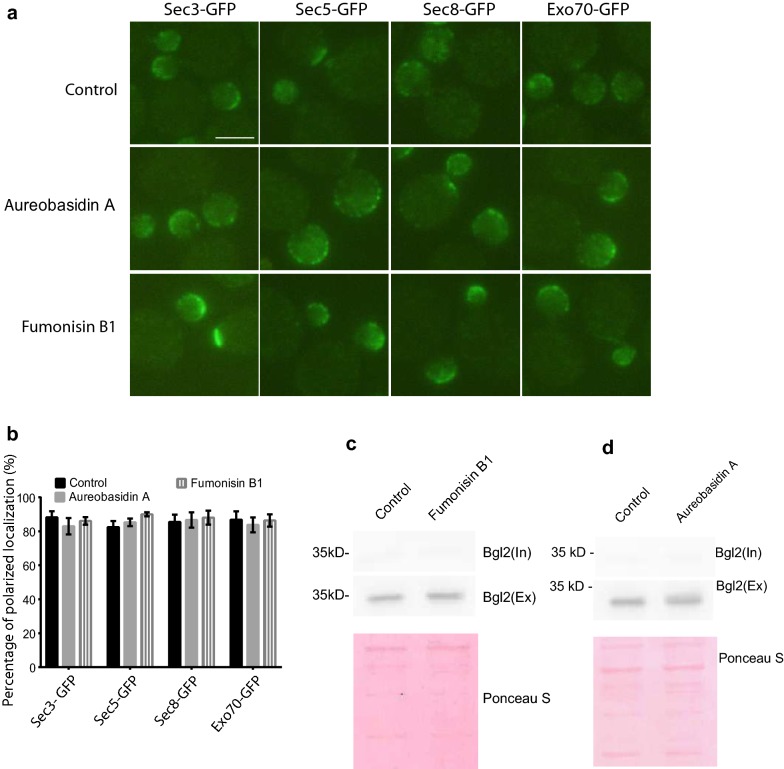
The synthesis of ceramides and complex sphingolipids is not required for exocytosis.** a** Localization of the exocyst components in cells treated with either Aureobasidin A or Fumonisin B1. Wild-type cells harboring *SEC3-GFP*, *SEC5-GFP*, *SEC8-GFP* or *EXO70-GFP* on the chromosome were grown to early log phase in YPD liquid medium overnight at 25 °C. The cells were then treated with 10 μg/ml Aureobasidin A or 500 μM Fumonisin B1 for 120 min. 2 µl of the suspension was dropped onto the microslide for microscopy. Scale bar, 2 μm. **b** Statistical analysis was performed for the percentage of polarized exocyst localization in each yeast strains **a**. n = 3. *p < 0.01. **c** Bgl2 secretion in wild type cells treated with or without Aureobasidin A. Wild-type cells were grown to early log phase in YPD liquid medium overnight at 25 °C. Aureobasidin A was added into the medium to a final concentration of 10 μg/ml, and the cells were incubated at 25 °C for 120 min. External and internal Bgl2 secretion was analyzed by western blot using anti-Bgl2 antibody. The total protein on PVDF membranes were stained with Ponceau S as loading control. **d** Bgl2 secretion in wild type cells treated with or without Fumonisin B1. Wild-type cells were grown to early log phase in YPD liquid medium overnight at 25 °C. Fumonisin B1 was added into the medium to a final concentration of 500 μM, and the cells were incubated at 25 °C for 120 min

Previous reports indicate exocytic vesicles contain large amount of ceramide and complex sphingolipids including IPC, MIPC, and M(IP)_2_C [20, 43], suggesting these sphingolipids may mediate exocytic secretion. To test this hypothesis, we examined the exocyst polarity and exocytic secretion in cells in which the synthesis of ceramides and complex sphingolipids were impaired with Fumonisin B1 and Aureobasidin A, respectively. As shown in Fig. 5a, b, both treatments of 500 μM Fumonisin B1 and 10 μg/ml Aureobasidin A did not affect the polarized localization of exocyst subunits of Sec3-GFP, Sec5-GFP, Sec8-GFP, and Exo70-GFP, even the cell growth was significantly inhibited at the same conditions (data not shown). We also measured the secretion of Bgl2, the cell wall endo-glucanase, in cells treated with either Fumonisin B1 or Aureobasidin A. As shown in Fig. 5c, d, similar to cells without treatment, there was no accumulation of internal Bgl2 found in the cytosol in cells treated with either Fumonisin B1 or Aureobasidin A. These results suggest that ceramides and complex sphingolipids are not required for exocytosis.

## Discussion

Sphingolipids are abundant components of eukaryotic membranes, where they play essential biological roles. Previous data showed that exocytic vesicles and the trans-Golgi network (TGN) contain large amount of sphingolipids, including ceramide, IPC, MIPC, and M(IP)_2_C [20], suggesting that these sphingolipids may mediate exocytosis. Several studies have suggested that sphingolipids may be involved in regulation of exocytosis [3, 17, 20, 21, 31, 37], but their roles in this critical cellular process is unclear. Although it has been reported that exocytosis is selectively blocked in *lcb1-100* cells at restrictive temperature, vesicle accumulation or secretory compartment enlargement had not been detected [17]. Because cells with a block in the secretory pathway usually accumulate secretory vesicles or enlarge secretory compartments such as elongated ER or Berkeley Bodies [35], the result that there was no accumulation of secretory vesicles or enlarged secretory compartments found in *lcb1-100* seems unusual and is controversial to the fact that exocytosis is selectively blocked in this mutant [17]. To confirm the requirement of sphingolipids for exocytosis, we treated *lcb1-100* mutant at a higher restrictive temperature of 39 °C, which has used to inhibit the Lcb1 enzyme activity in *lcb1-100* mutant strain [4, 19]. Under our experimental conditions, we detected block of exocytic secretion and accumulation of small secretory vesicles (Figs. 2, 4), indicating that sphingolipids are required for exocytosis. This conclusion is confirmed by the results that exocytic secretion was blocked in cells treated with myriocin (Fig. 2). The experimental conditions may contribute to the different electron microscopy results between our study and the previous report.

Although it has been reported that exocytic vesicles contain ceramides and complex sphingolipids including IPC, MIPC, and M(IP)_2_C [20], our results show that the exocyst polarity and exocytic secretion were not affected in cells defective in the synthesis of ceramides and complex sphingolipids (Fig. 5), suggesting that the exocytic secretion does not need the synthesis of ceramide and complex sphingolipids. Instead, our results indicate the delivery of ceramide and complex sphingolipids to plasma membrane relies on exocytosis. It has been suggested that sphingosines are produced in ER, and they have two roles in cells, either as a component in membranes or as transduction signal molecules [6, 7]. Because the *lcb1-100* mutant is unable to produce sphingoid bases in ER, we speculate that ER-to-Golgi exocytic vesicles contain sphingosines or the fusion of these vesicles with Golgi requires sphingosines. Further experiments are needed to confirm this speculation.

One interesting result found in our study is that sphingolipids regulate the subcellular localization of Sec3 (Figs. 1, 3). Unlike other exocyst subunits, Sec3 itself exists on the plasma membrane and does not rely on the transport of secretory vesicles to polarized growth sites [1, 13, 17, 26]. Currently, the regulators found to be able to control the polarized localization of Sec3 includes Cdk1, Cdc42, and Rho1 [10, 13, 28, 29, 49]. Therefore, the mispolarization of Sec3 in serine palmitoyl-transferase defecting cells may due to the composition change of sphingolipids in the plasma membrane or the activity reduction of Cdk1, Cdc42, and Rho1. Further experiments are required to explore how sphingolipids regulates the polarity of Sec3 at growth sites.

It has been reported that the polarity of the actin organization was disrupted in *lcb1-100* cells or in cells treated with myriocin [46]. Because the polarity of most exocyst subunits relies on the transport of post-Golgi secretory vesicles along actin cables to polarized growth sites [1, 13, 17, 26], one may argue that the mis-localization of Sec5-GFP, Sec8-GFP, and Exo70-GFP in *lcb1-100* cells or in cells treated with myriocin is an indirect effects of actin impairment. However, the electron microscopy results in our study show that the diameter of accumulated small vesicles is around 40 nm, which is very different from the size of post-Golgi secretory vesicles, 80–100 nm, suggesting the mis-localization of Sec5-GFP, Sec8-GFP, and Exo70-GFP in *lcb1-100* cells or in cells treated with myriocin is not an outcome of exocytic transport of Golgi apparatus to plasma membrane. Considering the size of small vesicles and the fact that the SPT enzyme is located in ER, we speculate that the impairment of exocytic transport from ER to Golgi apparatus led the mis-localization of Sec5-GFP, Sec8-GFP, and Exo70-GFP.

Although sphingolipids have long been considered as an intermediate in the process of sphingolipid metabolism, it was reported that the level of sphingolipid LCBs increased transiently and activated Pkh1/2 kinases by thermal stimulation [24]. Pkh1/2 are highly homologous to mammalian PDK1, and can activate the downstream AGC protein kinases, such as Ypk1/2 (gamma cell SGK1 homolog), Sch9 (homologous to mammalian cells Akt, PKB, S6K), and Pkc1 (mammalian cell PKC homologous), to regulate several biological processes including proliferation and aging [4–6, 27, 32]. Since sphingolipids are required for exocytic secretion, one would expect that Pkh1/2 and the downstream AGC kinases also involved in exocytosis. However, we found that the exocytic secretion is not affected in the mutants of these kinases (unpublished data), suggesting the sphingolipid LCB-Pkh1/2 pathway did not regulate exocytic secretion process. It seems that sphingolipids regulate exocytic secretion through controlling sphingolipid metabolism, but the possibility that sphingolipids regulate exocytosis through other signal pathway is not excluded.

## Conclusions

Our results suggest that sphingolipids are required for exocytosis. Mammals may use similar regulatory mechanisms because components of the exocytic secretion apparatus and signaling pathways are conserved.

## Methods

### Yeast strains and plasmids

Standard methods were used for yeast growth and genetic manipulations [42]. All strains used in this study are listed in Additional file 1: Table S1, and all plasmids used are listed in Additional file 1: Table S2. The plasmids were linearized by restriction enzymes and then used to transform yeast strains to make *GFP* tagged *SEC3*, *SEC5*, *SEC8*, and *EXO70* on the chromosome, respectively. Yeast transformation was performed using lithium acetate method [11]. Briefly, about 2 OD_600_ units of cells grown to early log phase were collected in a 1.5-ml centrifuge tube and washed twice with 1 ml of distilled water. To prepare the transformation reaction, 240 μl PEG 3350 (50% weight/volume), 36 μl 1.0 M LiAc, 5 μl 10 mg/ml ssDNA, and 0.1–10 μg DNA were added in order and the tube was vortexed until the cell pellet had been completely mixed. The suspended cells were incubated in a water bath pre-warmed to 42 °C for 30 min. After that, 200 μl of the transformation mix was plated onto a SD-Ura plate. The plates were incubated at 25 °C for 3–4 days to recover transformants.

### Bgl2 secretion

The classical Bgl2 secretion assay was used in this study with minor modifications [14]. 20 ml of yeast cells were grown to early log phase until OD_600_ is 0.6–1.0 at 25 °C. NaN_3_ and NaF were added directly to the culture at a final concentration of 10 mM each. 10 OD_600_ units of cells were collected, washed with cell wash buffer (20 mM Tris–HCl, pH 7.5, 10 mM NaN_3_, and 10 mM NaF). The cells were resuspended in 300 µl of spheroplast solution buffer (50 mM Tris–HCl, pH 7.5, 1.4 M sorbitol, 10 mM NaN_3_, 10 mM NaF, 30 mM 2-Mercaptoethanol, and 0.2 mg/ml Zymolyase) and incubated at 37 °C in a water bath for 30 min. The spheroplasts were pelleted gently by centrifuge at 2000 rpm for 5 min at 4 °C. 100 µl of supernatant was carefully transferred to a new tube and mixed with 20 µl of 6 × SDS loading buffer (300 mM Tris–HCl, pH 6.8, 600 mM dithiothreitol, 12% SDS, 0.6% bromophenol blue, and 60% glycerol). This is the external pool. The remaining supernatant was removed and the pellet (spheroplasts) was washed once with 1 ml of spheroplast wash buffer (50 mM Tris–HCl, pH 7.5, 1.4 M sorbitol, 10 mM NaN_3_, and 10 mM NaF) to remove residue external pool. The spheroplasts were dissolve in 300 µl of lysate buffer (20 mM Tris–HCl, pH 7.5, 100 mM NaCl, 2 mM MgCl_2_, 0.5% Triton X-100, and 1 × protease inhibitor cocktail from Roche Inc.) by leaving on ice for 10 min. The cell debris was removed after the sample was centrifuged at 13,000 rpm for 5 min at 4 °C. 100 µl of supernatant (lysates) was transferred to a new tube and mixed with 20 µl of 6 × SDS loading buffer. This is the internal pool. The internal pool and external pool samples were boiled at 95 °C for 5 min, loaded into 12% SDS-PAGE gel. Bgl2 was detected by Western blotting with an anti-Bgl2 rabbit polyclonal antibody (1:2,000 dilution). For temperature-sensitive mutants, the cells were grown at 25 °C or shifted to 39 °C for 60 min before being processed for the Bgl2 assay. For myriocin treatment, the cells were exposed to 10 µg/ml myriocin for 60 min before being processed for secretion assays.

### Invertase secretion

Invertase secretion was examined as described previously [35]. 20 ml of yeast cells were grown to early log phase at 25 °C until OD_600_ is 0.6–1.0. One OD_600_ cells were transferred to a new tube and washed with 1 ml of ice cold 1 mM NaN3. The cells were then resuspended in 1 ml YP plus glucose medium (1% Bacto-yeast, 2% Bactopeptone, and 0.1% glucose) and incubated at 25 °C for 1 h to induce invertase expression. After 1 h of incubation, the cells were collected and washed once with 1 ml 10 mM NaN3. The cells were resuspended in 1 ml 10 mM NaN3 and kept on ice. The external invertase was measured directly on the whole intact cells, whereas the internal invertase was measured after preparation of lysates. 0.5 ml of cells were removed and mixed with 0.5 ml of 2 × spheroplast cocktail mix (2.8 M sorbitol, 0.1 M Tris–HCl pH 7.5, 10 mM NaN_3_, 0.4% 2-Mercaptoethanol, and 10 µg/ml Zymolyase-100 T). The cells were incubated in water bath at 37 °C for 45 min. The spheroplasts were collected and the supernatant was removed carefully without disturbing the pellet. The spheroplasts were dissolved at 4 °C in 0.5 ml 0.5% Triton X-100. The invertase assay was performed in 13 × 100 mm glass tubes. 20 µl of sample was placed in the tube and 80 µl of 50 mM NaAc, pH 5.1, was added. Then 25 µl of 0.5 M sucrose was added and the tube was incubated at 37 °C for 30 min. 150 µl of 0.2 M K_2_HPO_4_ was added and the tube was placed on ice to stop the reaction. The sample was boiled for 3 min and put on ice. One ml of assay mix was added (0.1 M KP_i_ buffer, pH 7.0, 10 U/ml glucose oxidase, 2.5 µg/ml peroxidase, 150 µg/ml O-dianisidine, and 20 µM N-Ethylmaleimide) and the sample was incubated at 37 °C for 30 min. One ml of 6 N HCl was added into the tube and the value of OD_540_ was measured by the Spectrophotometer (SmartSpec 3000; Bio-Rad Laboratories). For temperature-sensitive mutants, the cells were grown at 25 °C or shifted to 39 °C for 60 min and then were grown in low-glucose medium (0.1% glucose) at the same temperature for 1 h. For cells treated with myriocin, the cells were treated with 10 µg/ml myriocin for 60 min before being processed for secretion assays.

### Fluorescent microscopy

Chromosomal tagging of *SEC3*, *SEC5*, *SEC8*, and *EXO70* with Green Fluorescence Protein (*GFP*) was performed as previously described [47]. Cells were grown to log phase at 25 °C, then shifted to 39 °C or treated with myriocin for 60 min. The cells were then fixed for microscopy as described previously [23]. Immuno-fluorescence staining of yeast cells was performed as previously described [26]. Rabbit anti-Sec4 was used at a 1:1,000 dilution followed by a secondary AlexaFluor594-conjugated goat anti-rabbit IgG antibody (Molecular Probes). Fluorescent microscopy was conducted with a fluorescence microscope (BX53; OLYMPUS) equipped with a Plan-Apochromat × 00, 1.40 NA oil immersion objective lens. Images were taken using cellSens Standard software (OLYMPUS). Images were processed using Adobe Photoshop CC 2019 software, and statistical analysis was performed using GraphPad Prism 7.0 software.

### Electron microscopy

The electron microscopy (EM) was performed as the previous study with minor modifications [8]. Cells were collected by vacuum filtration using a 0.45 µm nitrocellulose membrane and were fixed for 1 h at room temperature in 0.1 M cacodylate, pH 7.4, 3% formaldehyde, 1 mM MgCl_2_, and 1 mM CaCl_2_. The cells were spheroplasted and fixed with 1% glutaraldehyde (in PBS, pH 7.4) at 4 °C overnight. The spheroplasts were washed in 0.1 M cacodylate buffer and were postfixed twice with ice-cold 0.5% OsO_4_ and 0.8% potassium ferrocyanide for 10 min each. After dehydration and embedding in Spurr’s epoxy resin (Polysciences, Inc.), thin sections were cut and transferred onto 600 mesh uncoated copper grids (Ernest Fullam, Inc.) and were post-stained with uranyl acetate and lead citrate. Cells were observed on a transmission electron microscope (Model 1010; JEOL) at ×100,000 magnification.

### Statistical analysis

Statistical analysis was performed using Graphpad Prism 7.0 software. One-way ANOVO was used for univariate analysis. p < 0.05 was considered statistically significant.

## Supplementary information

**Additional file 1: Figure S1.** The amount of the GFP tagged exocyst subunits did not differ significantly change in lcb1-100 mutant and cells treated with myriocin. (A) The yeast strains harboring SEC3-GFP, SEC5-GFP, SEC8-GFP or EXO70-GFP on the chromosome were grown to early log phase in YPD liquid medium overnight at 25°C. The cells were then shifted from 25℃ to 39℃ for 60 min. Total protein was extracted and Western blots were performed using rabbit anti-GFP antiby (1:1000 dilution). Adh1 was used as a loading control (rabbit anti-Adh1 antibody). (B) Wild-type cells harboring SEC3-GFP, SEC5-GFP, SEC8-GFP or EXO70-GFP on the chromosome were grown to early log phase in YPD liquid medium overnight at 25°C. The cells were then treated with or without 10μg/ml myriocin for 60 min. Total protein was extracted and Western blots were performed using rabbit anti-GFP antiby (1:1000 dilution). Adh1 was used as a loading control (rabbit anti-Adh1 antibody).** Figure S2.** Cell viability of wild type and lcb1-100 mutant cells at a restrictive temperature. (A) lcb1-100 mutant is temperature sensitive. Wild type and lcb1-100 mutant cells were grown to log phase in YPD at 25°C, and equal densities of wild-type or lcb1-100 mutant cells were spotted in 10-fold serial dilutions from left to right on YPD plate, and then incubated at different temperatures for 3 days. (B) Viability of lcb1-100 mutant cells at restrictive temperature. Wild-type and lcb1-100 mutant cells were grown to early log phase in YPD liquid medium overnight at 25°C and then shift to 39°C for 60 min. The cells were stained with 4% Loeffler’s methylene blue solution for 10min at room temperature. Cells were washed with PBS buffer and examined by microscopy. Cells stained with blue color are not viable. (C) Statistical analysis of cell viability in wild type and lcb1-100 mutant cells in (B). n=3. Three independent experiments were performed. *, p<0.01.** Figure S3.** Cell viability of wild type cells treated with or without myriocin. (A) Wild-type and lcb1-100 mutant cells were grown to early log phase in YPD liquid medium overnight at 25°C. Myriocin was added into the medium to a final concentration of 10μg/ml, and the cells were incubated at 25°C for 60 min. The cells were collected and stained with 4% Loeffler’s methylene blue solution for 10 min at room temperature. Cells were washed with PBS buffer and examined by microscopy. Cells stained with blue color are not viable. (B) Statistical analysis of viability of wild type cells treated with myriocin in (A). n=3. Three independent experiments were performed. *, p<0.01.** Table S1.** Yeast strains used in this study. **Table S2.** Plasmids used in this study.

## Data Availability

The data and materials used and/or analyzed during the current study are available from the corresponding author on reasonable request.

## Abbreviations

EM: Electron microscopy
GFP: Green fluorescent protein
IPC: Inositolphosphoryl ceramide
LCB: Long-chain base
MIPC: Mannosyl-inositolphosphoryl ceramide
M(IP)_2_C: Mannosyl-diinositolphosphoryl ceramide
SNARE: Soluble N-ethylmaleimide-sensitive factor activating protein receptor
SONC: Saturated overnight culture
SPT: Serine palmitoyl-transferase
TGN: Trans-Golgi network
WT: Wild type
Ve: Vesicle
