# Supplementary material for: Sphingolipids are required for exocyst polarity and exocytic secretion in Saccharomyces cerevisiae
Source: Cell Biosci. 2020 Mar 30;10:53. doi: 10.1186/s13578-020-00406-2 (PMC7106735; doi:10.1186/s13578-020-00406-2)
Supplement: Supplementary file 1 — Additional file 1: Figure S1. The amount of the GFP tagged exocyst subunits did not differ significantly change in lcb1-100 mutant and cells treated with myriocin. (A) The yeast strains harboring SEC3-GFP, SEC5-GFP, SEC8-GFP or EXO70-GFP on the chromosome were grown to early log phase in YPD liquid medium overnight at 25°C. The cells were then shifted from 25℃ to 39℃ for 60 min. Total protein was extracted and Western blots were performed using rabbit anti-GFP antiby (1:1000 dilution). Adh1 was used as a loading control (rabbit anti-Adh1 antibody). (B) Wild-type cells harboring SEC3-GFP, SEC5-GFP, SEC8-GFP or EXO70-GFP on the chromosome were grown to early log phase in YPD liquid medium overnight at 25°C. The cells were then treated with or without 10μg/ml myriocin for 60 min. Total protein was extracted and Western blots were performed using rabbit anti-GFP antiby (1:1000 dilution). Adh1 was used as a loading control (rabbit anti-Adh1 antibody). Figure S2. Cell viability of wild type and lcb1-100 mutant cells at a restrictive temperature. (A) lcb1-100 mutant is temperature sensitive. Wild type and lcb1-100 mutant cells were grown to log phase in YPD at 25°C, and equal densities of wild-type or lcb1-100 mutant cells were spotted in 10-fold serial dilutions from left to right on YPD plate, and then incubated at different temperatures for 3 days. (B) Viability of lcb1-100 mutant cells at restrictive temperature. Wild-type and lcb1-100 mutant cells were grown to early log phase in YPD liquid medium overnight at 25°C and then shift to 39°C for 60 min. The cells were stained with 4% Loeffler’s methylene blue solution for 10min at room temperature. Cells were washed with PBS buffer and examined by microscopy. Cells stained with blue color are not viable. (C) Statistical analysis of cell viability in wild type and lcb1-100 mutant cells in (B). n=3. Three independent experiments were performed. *, p<0.01. Figure S3. Cell viability of wild type cells treated with or without [file 13578_2020_406_MOESM1_ESM.pdf]

Fig. S1

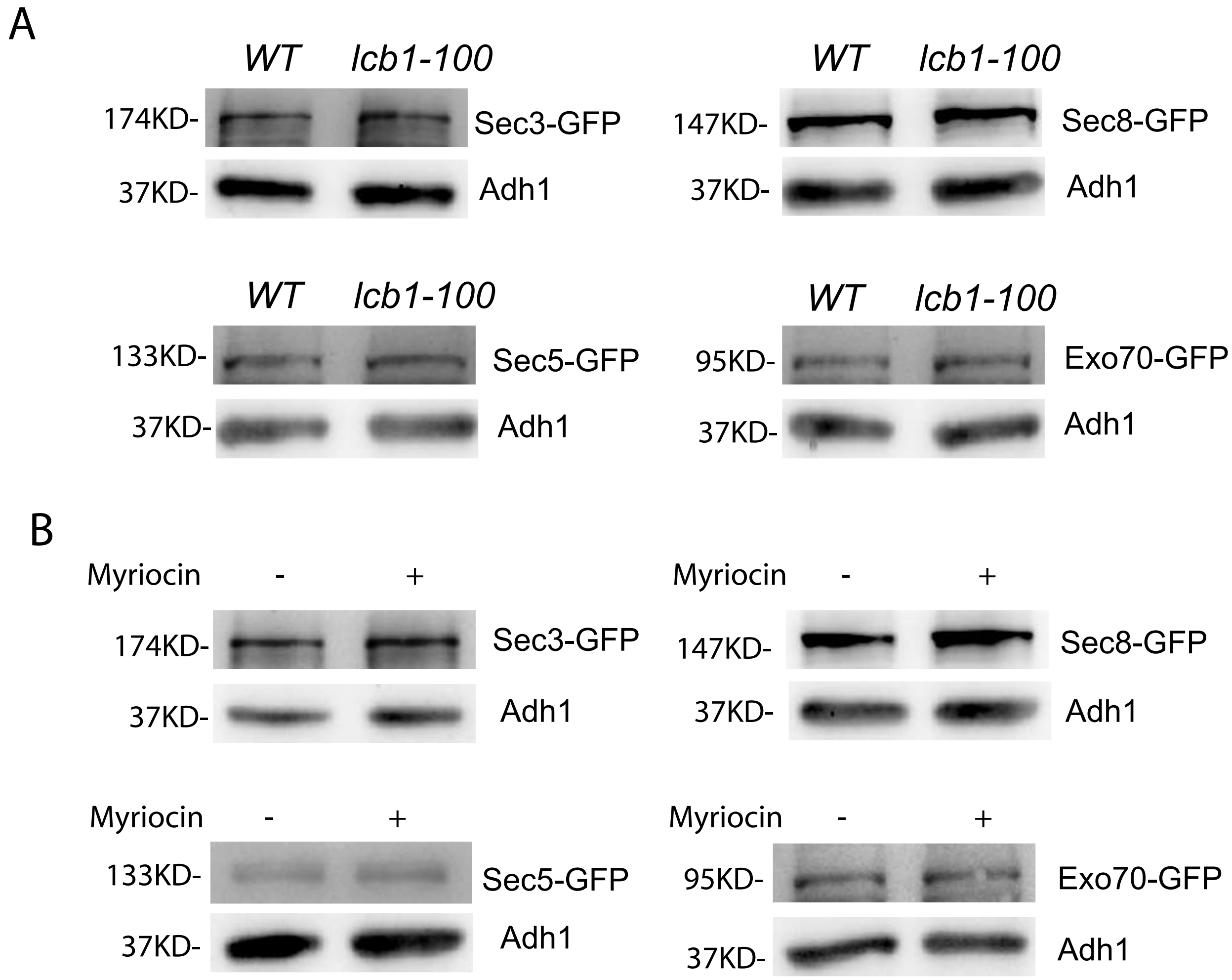
A
WT
lcb1-100
174KD-
Sec3-GFP
37KD-
Adh1
WT
lcb1-100
147KD-
Sec8-GFP
37KD-
Adh1
WT
lcb1-100
133KD-
Sec5-GFP
37KD-
Adh1
WT
lcb1-100
95KD-
Exo70-GFP
37KD-
Adh1
B
Myriocin - +
174KD-
Sec3-GFP
37KD-
Adh1
Myriocin - +
147KD-
Sec8-GFP
37KD-
Adh1
Myriocin - +
133KD-
Sec5-GFP
37KD-
Adh1
Myriocin - +
95KD-
Exo70-GFP
37KD-
Adh1

Fig. S2

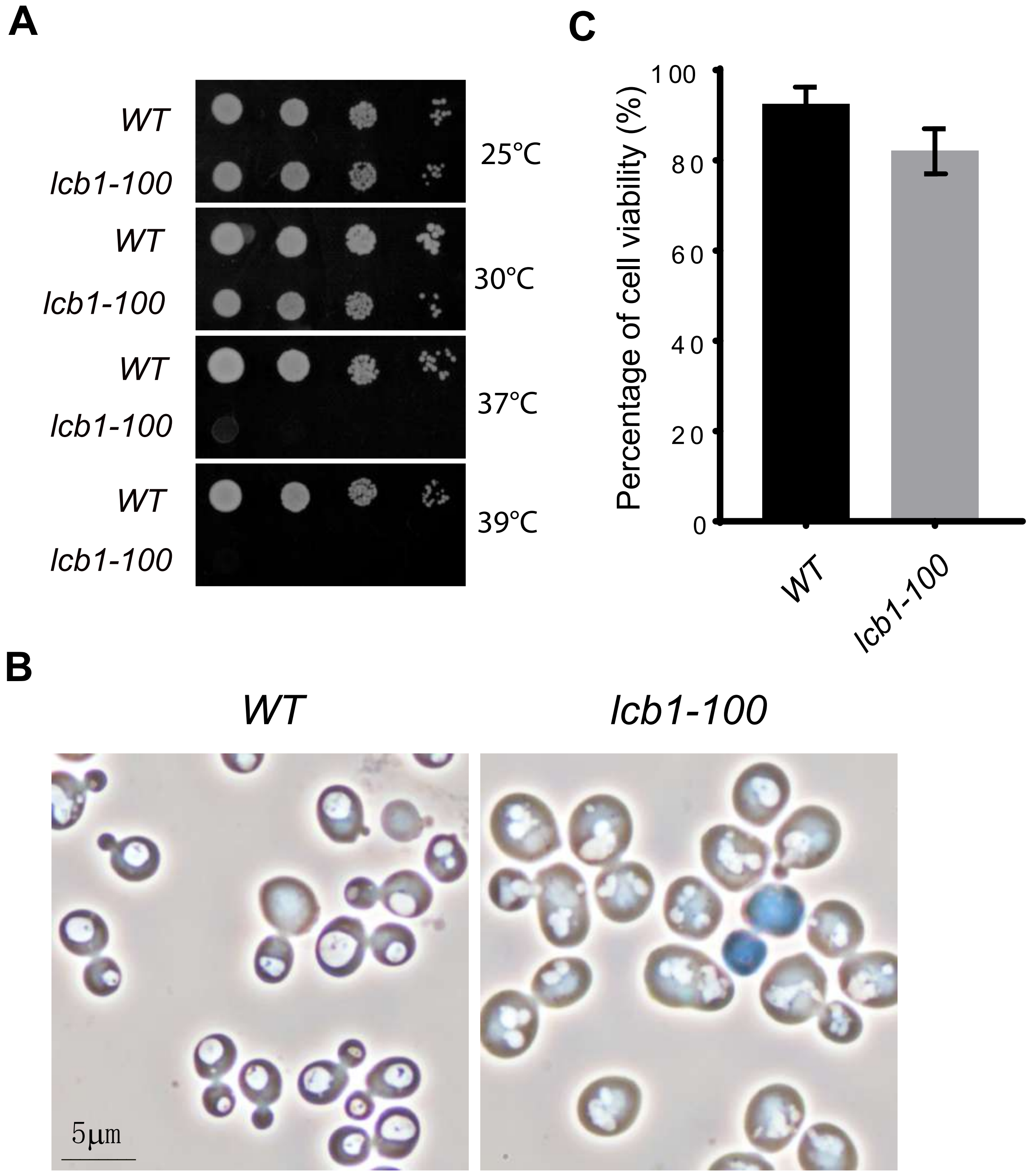
A
WT
lcb1-100
25°C
WT
lcb1-100
30°C
WT
lcb1-100
37°C
WT
lcb1-100
39°C
C
Percentage of cell viability (%)
100
80
60
40
20
0
WT
lcb1-100
B
WT
lcb1-100
5μm

Fig. S3

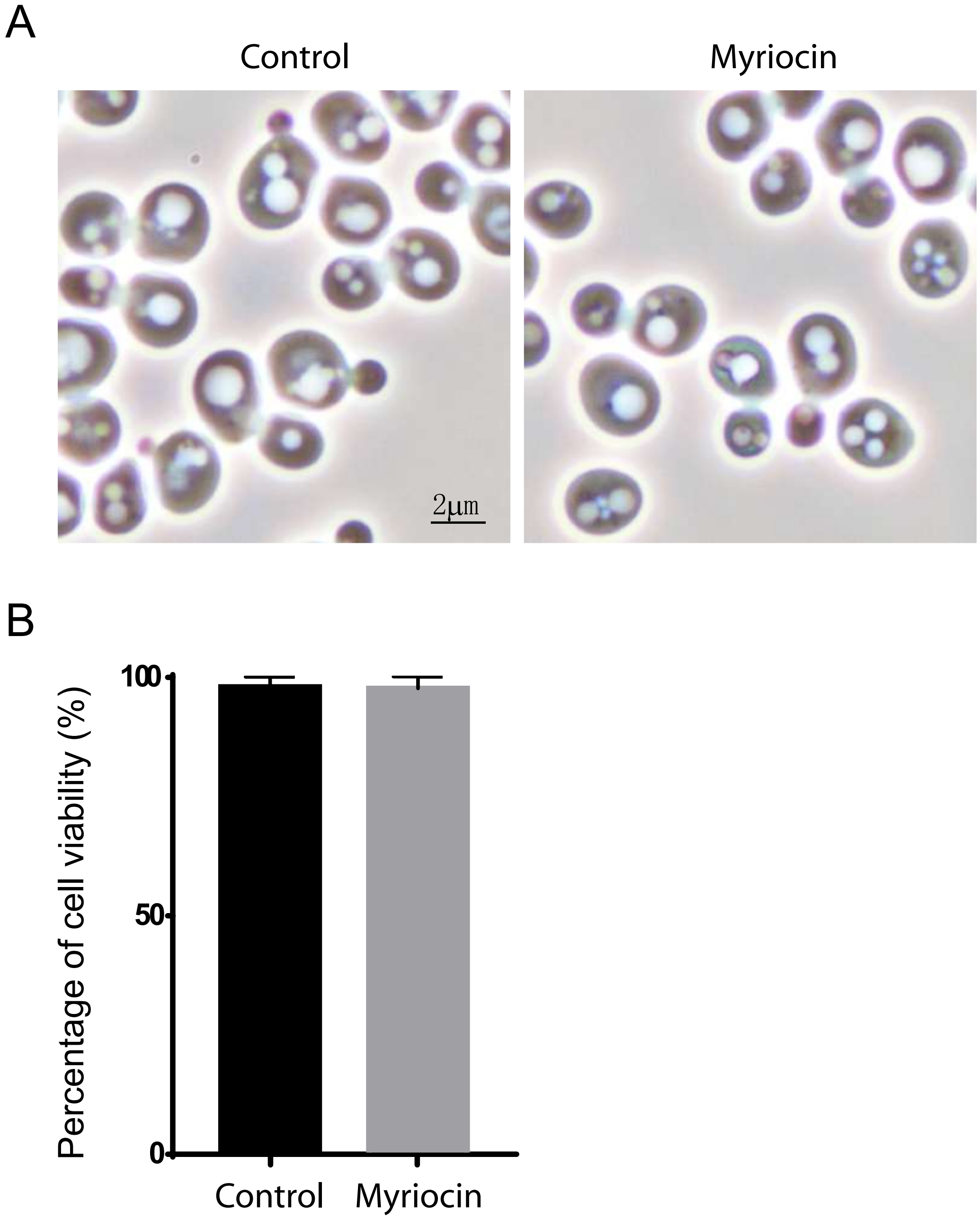

A
Control
Myriocin
2μm
B
Percentage of cell viability (%)
100
50
0
Control
Myriocin

Table S1. Yeast strains used in this study

Strain	Genotype	Reference
RH1800	*MATa, leu2, his4, ura3, bar1*	Raths *et al.*, 1993
RH3809	*MATa, his4, end8, ura3, leu2, bar1, lcb1-100*	Zanolari *et al.*, 2000

Table S2. Plasmids used in this study.

**Plasmid**	**Description**	**Reference**
pG1269	*pRS306-SEC3-GFP*, *URA3*, BstE II digestion for integration into *SEC3* locus.	Zhang *et al.*, 2008
NRB881	*pRS306-SEC5-GFP*, *URA3*, Afl II digestion for integration into *SEC5* locus.	Liu *et al.*, 2018
NRB885	*pRS306-SEC8-GFP*, *URA3*, Bgl II digestion for integration into *SEC8* locus.	Liu *et al.*, 2018
NRB880	*pRS306-EXO70-GFP*, *URA3*, BstE II digestion for integration into *EXO70* locus.	Liu and Novick, 2014
